# Supplementary material for: A functional SNP rs895819 on pre-miR-27a is associated with bipolar disorder by targeting NCAM1
Source: Commun Biol. 2022 Apr 4;5:309. doi: 10.1038/s42003-022-03263-6 (PMC8980034; doi:10.1038/s42003-022-03263-6)
Supplement: Supplementary file 2 — Supplementary Information (new) [file 42003_2022_3263_MOESM2_ESM.pdf]

Supplementary Figures

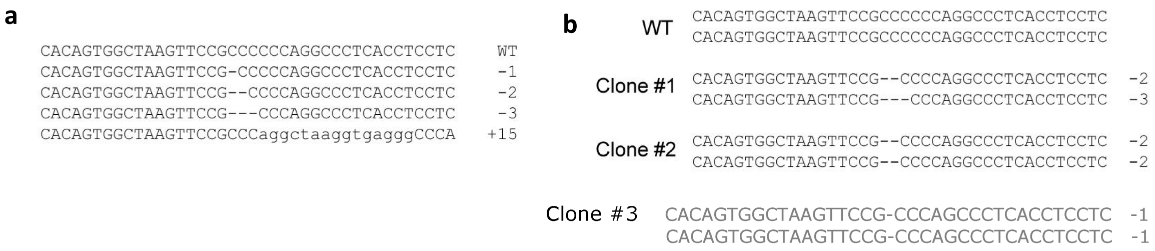

Supplementary Figure 1. Construction of miR-27a knockout cell line. a. Validation of miR-27-targeted sgRNA. **b.** Clone #1, Clone #2, Clonal analyses of gene-edited cells in astrocytoma U251. Clone #3, Clonal analyses of gene-edited cells in neuroblastoma SH-SY5Y.

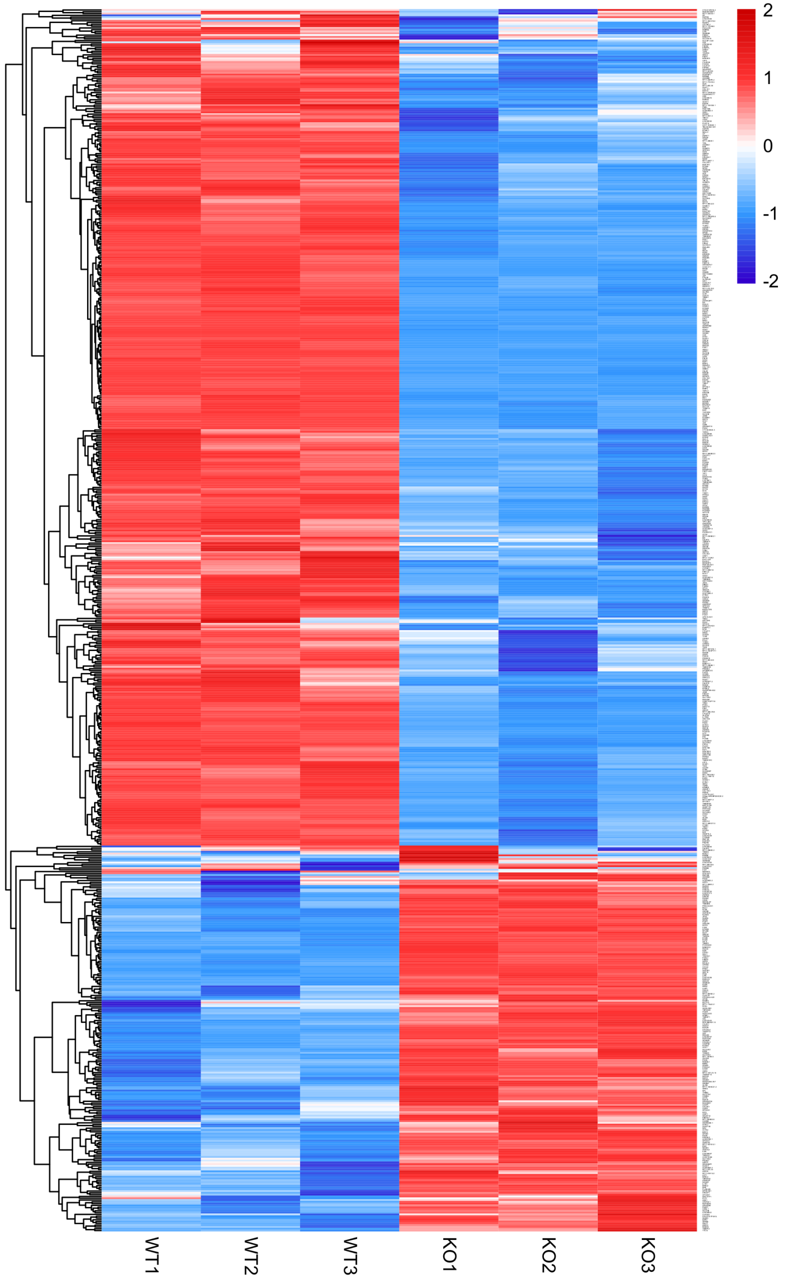
2
1
0
-1
-2
WT1
WT2
WT3
KO1
KO2
KO3

Supplementary Figure 2. Cluster analyses of differentially expressed genes.

The results are shown as heat map. Z score was used to normalize the value of FPKM (Fragments per Kilobase Million). WT1, WT2, WT3 indicates three samples of U251 wildtype. KO1, KO2, KO3 indicates three sample of miR-27a KO in U251. Each small grid represents each gene, and the shade of color represents the expression level of this gene. The larger the expression level is, the darker the color will be (red is up-regulated, blue is down-regulated). Each row represents the expression of each gene in a different sample, and each column represents the expression of all genes in each sample. The left tree represents the clustering analysis results of different genes from different samples.

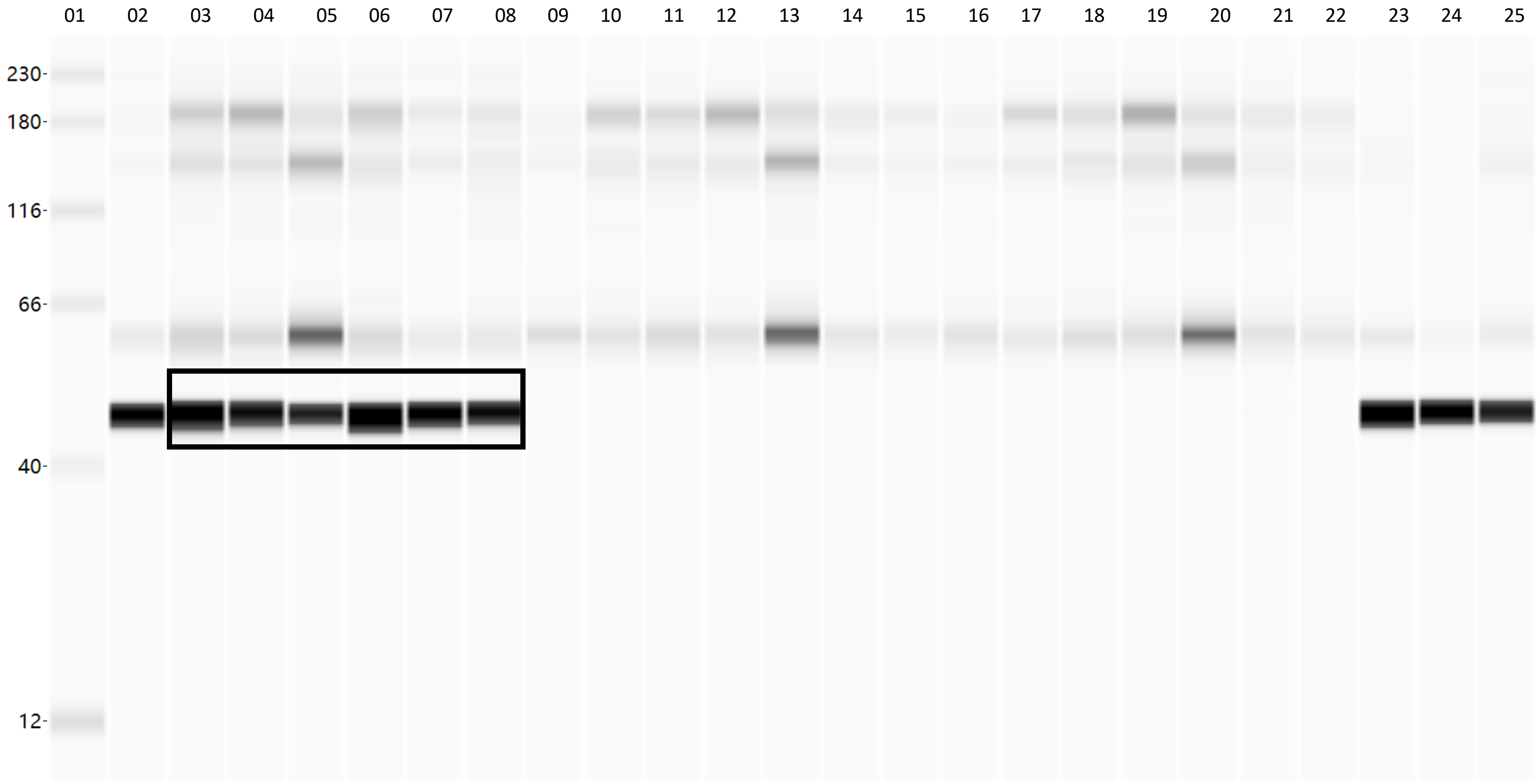

Supplementary Figure 3. Uncropped gels of Fig. 4b for β-actin in U251.

Lane 01 is protein marker. The stripes in black frame from lane 03 to lane 08 denote mimic NC, mimic 30 pmol, mimic 90 pmol, inhibitor NC, inhibitor 30 pmol and inhibitor 90 pmol. The observed strips are β-actin at 42 KDa in U251.

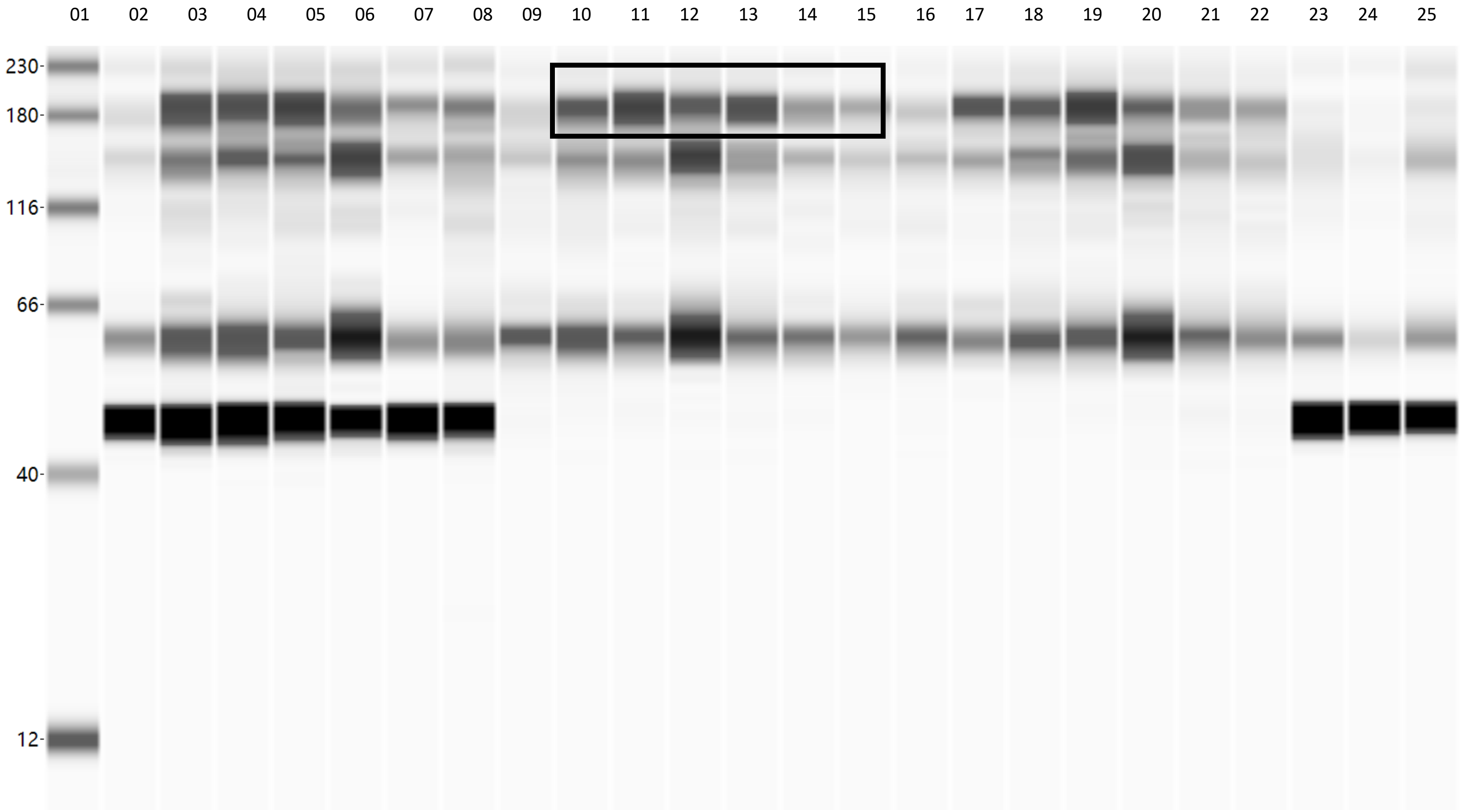

Supplementary Figure 4. Uncropped gels of Fig. 4b for NCAM1 in U251.

Lane 01 is protein marker. The stripes in black frame from lane 10 to lane 15 denote mimic NC, mimic 30 pmol, mimic 90 pmol, inhibitor NC, inhibitor 30 pmol and inhibitor 90 pmol. The observed strips are NCAM1 at 180 KDa in U251.

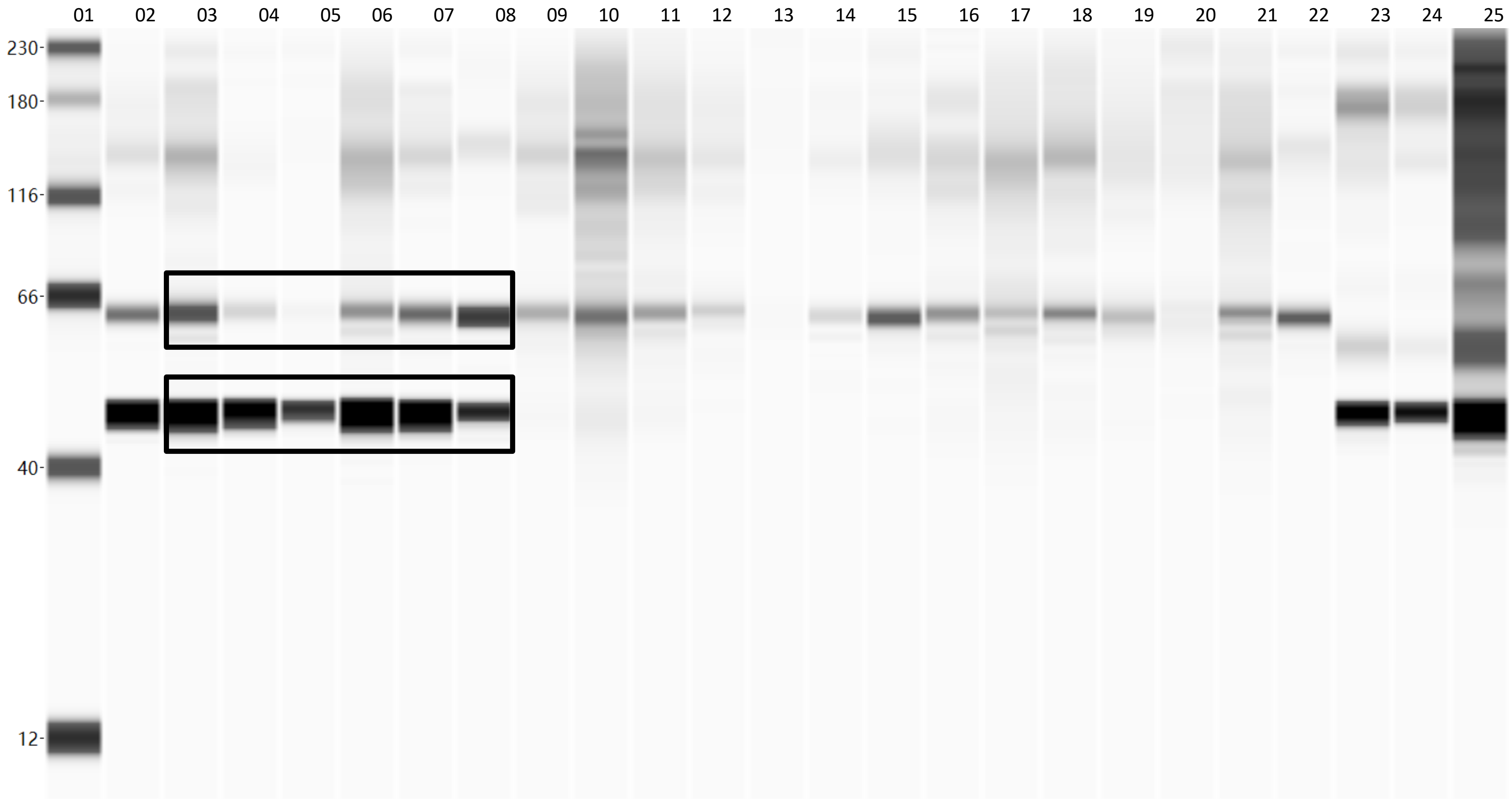

Supplementary Figure 5. Uncropped gels of Fig. 4b for NCAM1 and β-actin in NPC.

Lane 01 is protein marker. The stripes in black frame from lane 03 to lane 08 denote mimic NC, mimic 1 pmol, mimic 10 pmol, inhibitor NC, inhibitor 10 pmol and inhibitor 10 pmol. The observed strips are NCAM1 at 60 KDa, β-actin at 42 KDa in NPC.

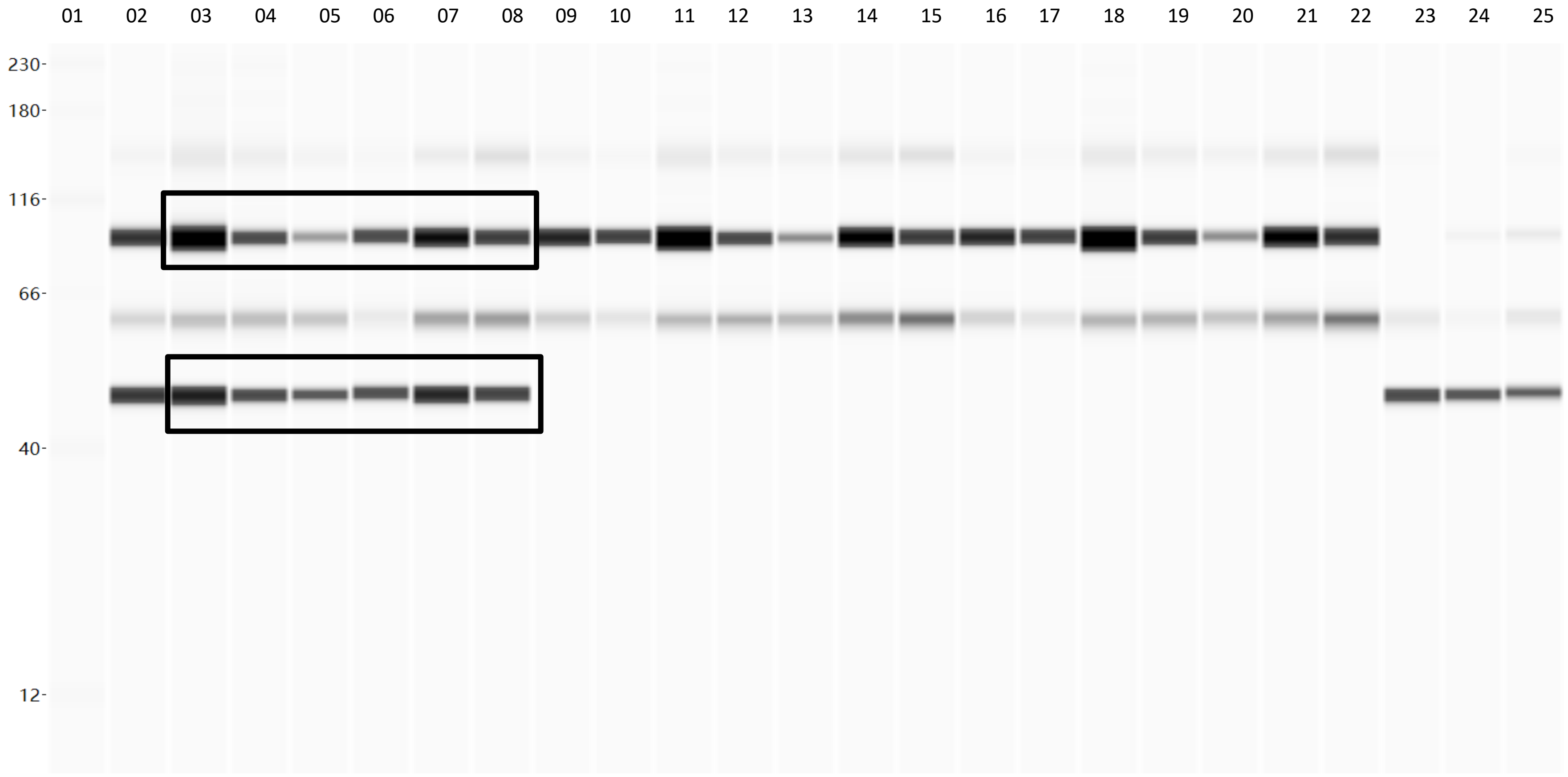

Supplementary Figure 6. Uncropped gels of Fig. 4c for ICAM1 and β-actin in U251.

Lane 01 is protein marker. The stripes in black frame from lane 03 to lane 08 denote mimic NC, mimic 30 pmol, mimic 90 pmol, inhibitor NC, inhibitor 30 pmol and inhibitor 90 pmol. The observed strips are ICAM1 at 95 KDa, β-actin at 42 KDa in U251.

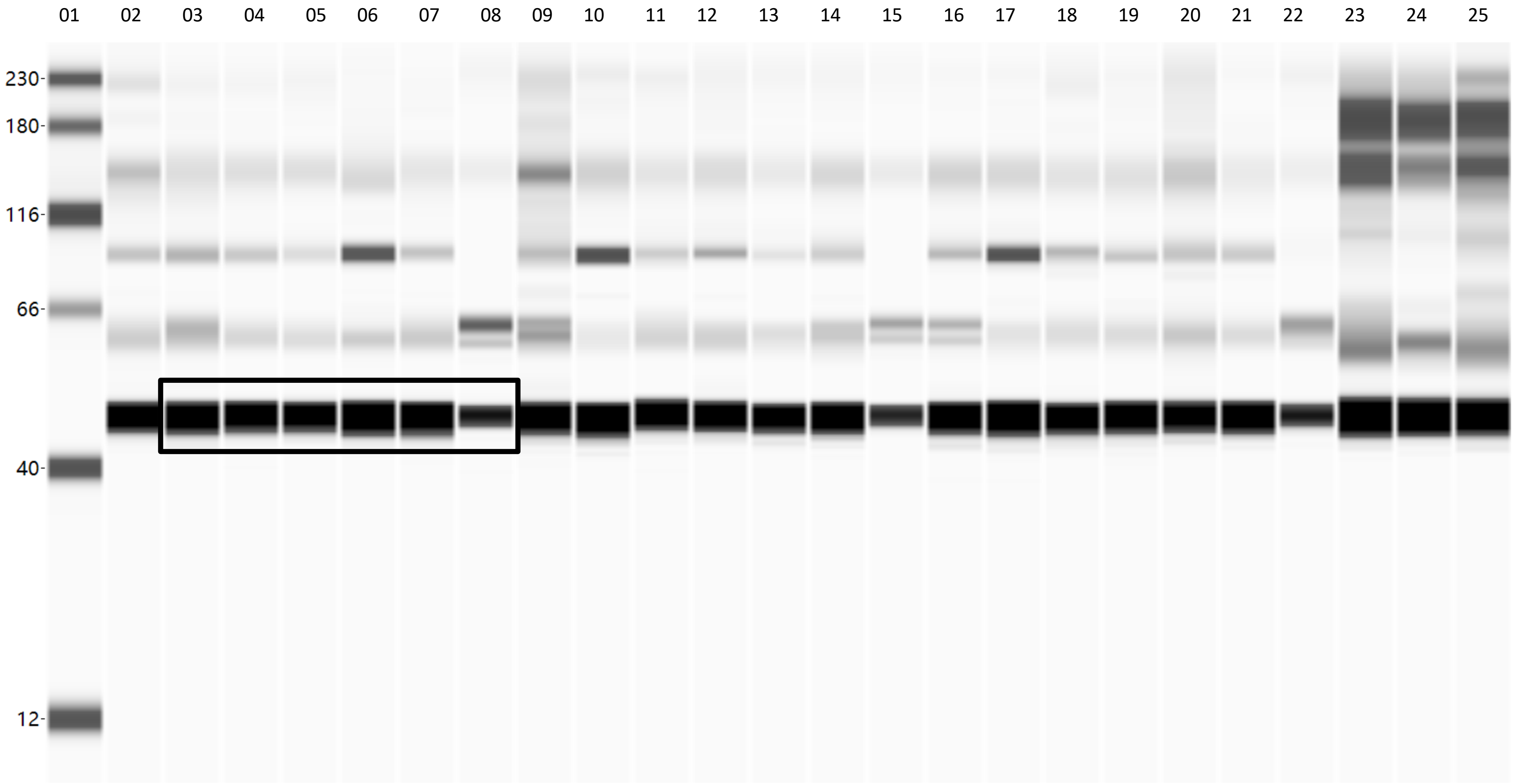

Supplementary Figure 7. Uncropped gels of Fig. 4c for β-actin in NPC.

Lane 01 is protein marker. The stripes in black frame from lane 03 to lane 08 denote mimic NC, mimic 1 pmol, mimic 10 pmol, inhibitor NC, inhibitor 1 pmol and inhibitor 10 pmol. The observed strips are β-actin at 42 KDa in NPC.

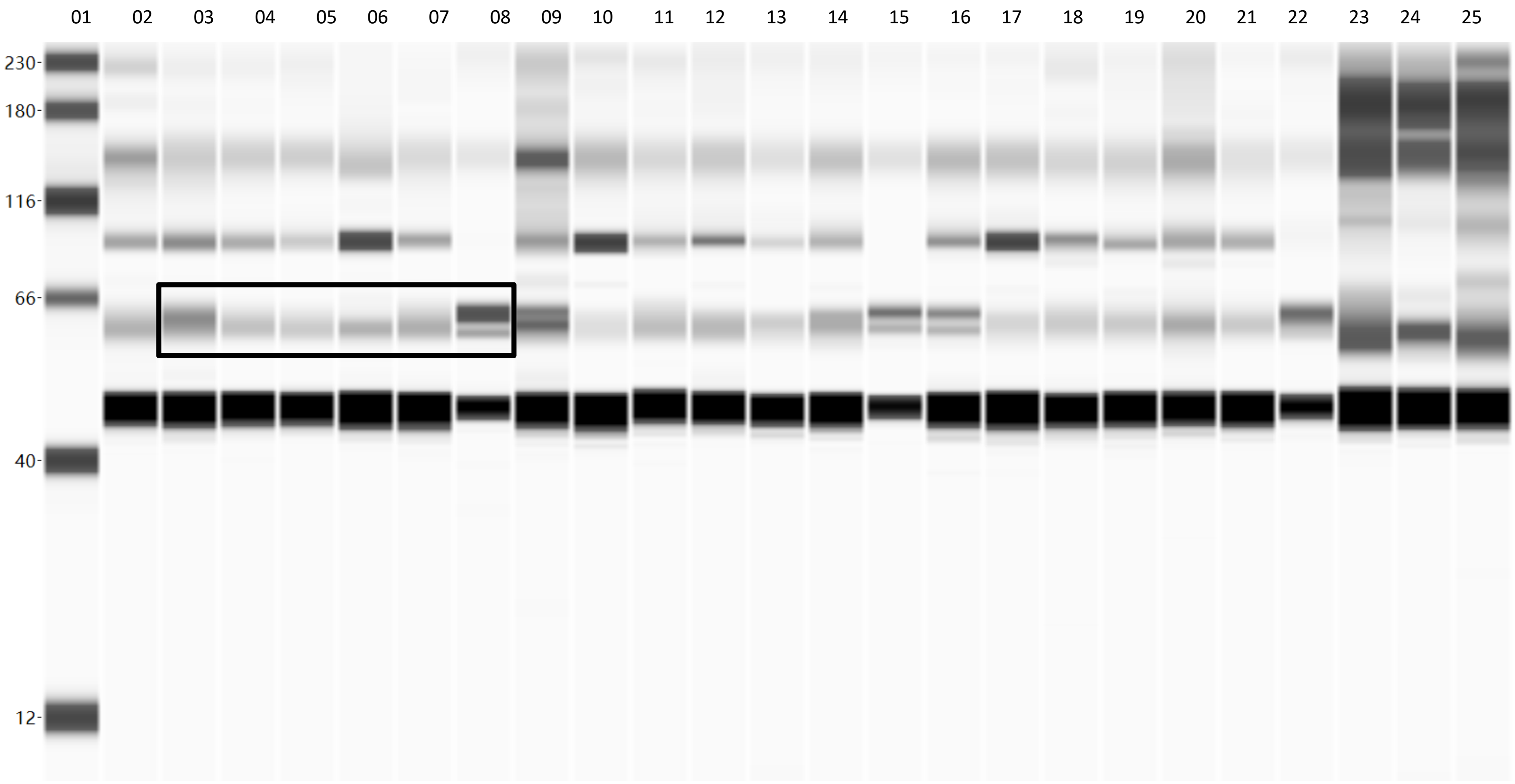

Supplementary Figure 8. Uncropped gels of Fig. 4c for ICAM1 in NPC.

Lane 01 is protein marker. The stripes in black frame from lane 03 to lane 08 denote mimic NC, mimic 1 pmol, mimic 10 pmol, inhibitor NC, inhibitor 1 pmol and inhibitor 10 pmol. The observed strips are ICAM1 at 62 KDa in NPC.

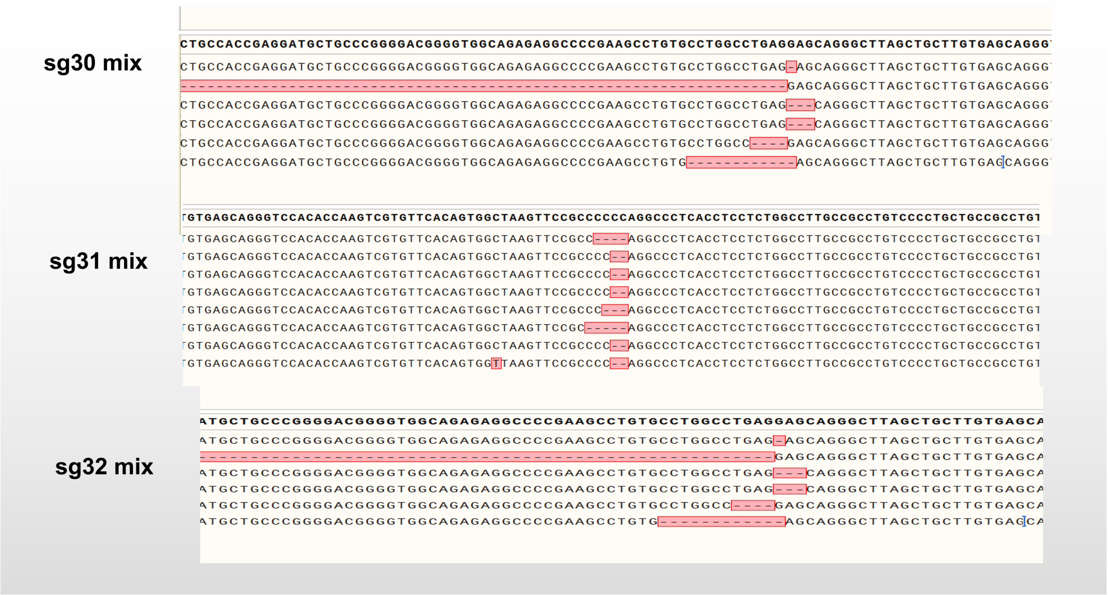

Supplementary Figure 9. The validation of miR-27a KO by direct sequencing. Sg30 mix, sg31 mix and sg32 mix were constructed to knock out miR-27a in astrocytoma U251 by CRISPR-Cas9 method using three different sgRNAs to minimize the off-target effect. The three multi-clones of miR-27 KO cell lines were validated by direct sequencing.

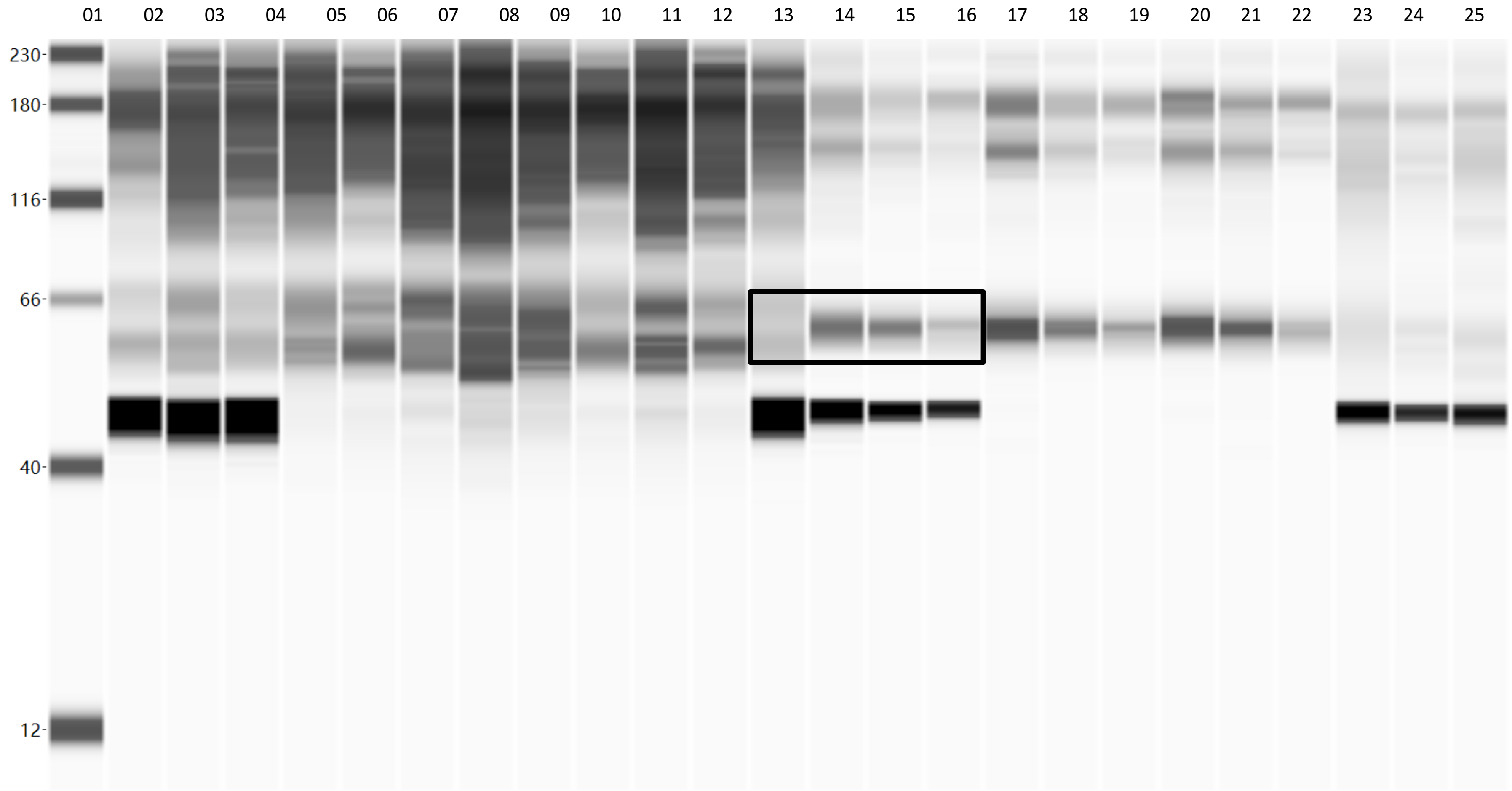

Supplementary Figure 10. Uncropped gels of Fig. 4f for NCAM1 in U251.

Lane 01 is protein marker. The stripes in black frame from lane 13 to lane 16 denote WT, sg30-mix, sg31-mix, and sg32-mix. The observed strips are NCAM1 at 60 KDa in U251.

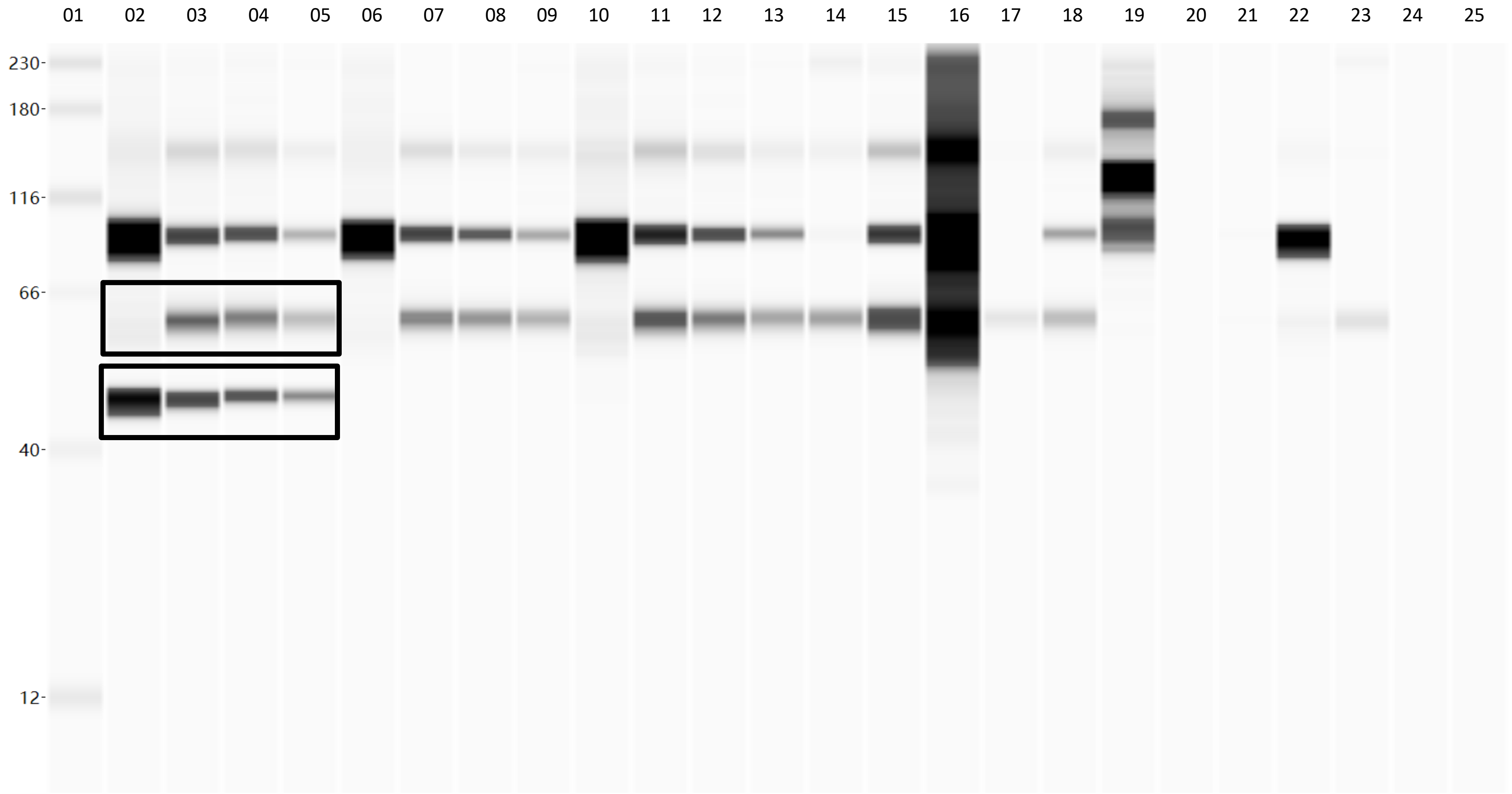

Supplementary Figure 11. Uncropped gels of Fig. 4f for ICAM1and β-actin in U251.

Lane 01 is protein marker. The stripes in black frame from lane 02 to lane 05 denote WT, sg30-mix, sg31-mix and sg32-mix. The observed strips are ICAM1 at 62 KDa , β-actin at 42 KDa in U251.

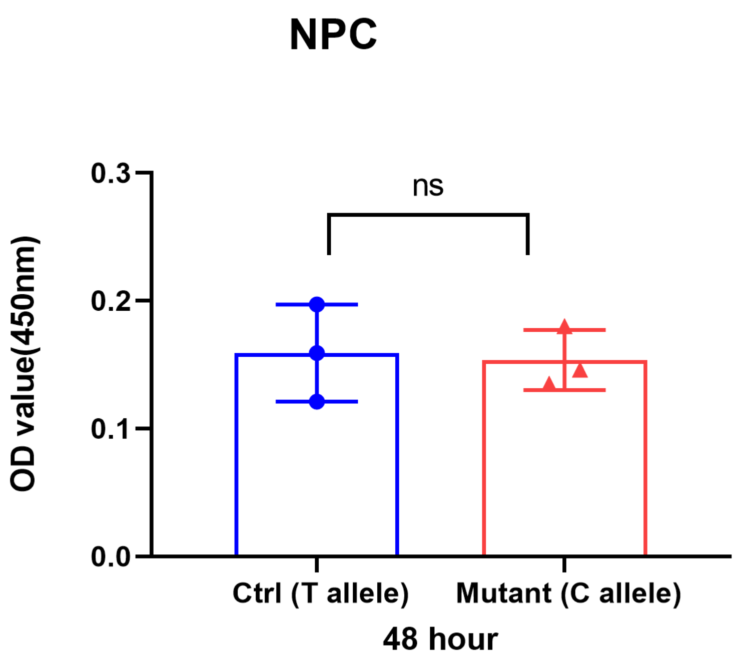

Supplementary Figure 12. The C mutant of miR-27a has no effect on NPC proliferation. Proliferation of NPC Ctrl (T allele) and NPC Mutant (C allele) was examined using the CCK8 assay at 48 hours. Unpaired t-test was performed between two groups with three replicates（n=3）for each group. Results were expressed as the mean ± SD. P<0.05 as a sign of significance；n.s. not significant, P>0.05.

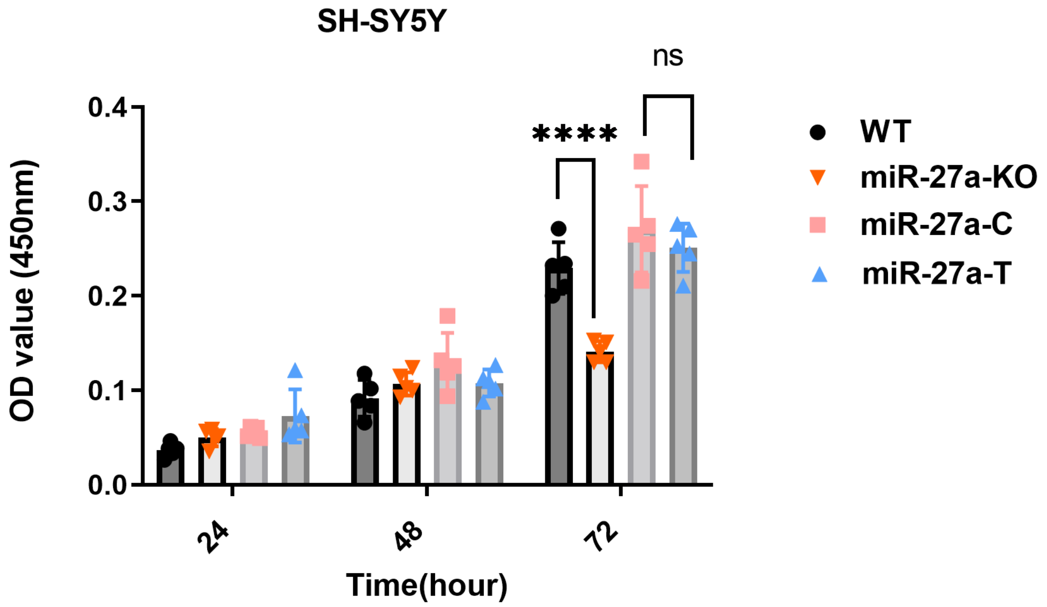

Supplementary Figure 13. The knockout of miR-27a inhibit the growth of SH-SY5Y.

Proliferation of several cell lines (WT, C allele, T allele and miR-27a KO in SH-SY5Y) was examined using the CCK8 assay at 24, 48 and 72 hours, respectively. Significant difference between WT and KO were analyzed by one-way ANOVA; n=5 (*, P < 0.05, **, P < 0.01; ***, P < 0.001) .n.s. not significant, P>0.05. Data were expressed as the mean ± SD.

Supplementary Tables

Supplementary Table 1. Primers and RNA sequences used in this study

Name	Sequence	Usage
GAPDH-F	5’-GCCAGCCTCGTCTCATAGAC-3’	Internal reference for RT-PCR
GAPDH-R	5’-AGTGATGGCATGGACTGTGG-3’	Internal reference for RT-PCR
NCAM1-F	5’-GGCATTTACAAGTGTGTGGTT-3’	RT-qPCR of NCAM1
NCAM1-R	5’-TTGGCGCATTCTTGAACATGA-3’	RT-qPCR of NCAM1
IL6R-F	5’-CCCCTCAGCAATGTTGTTTGT-3’	RT-qPCR of IL6R
IL6R-R	5’-CTCCGGGACTGCTAACTGG-3’	RT-qPCR of IL6R
CSF1-F	5’-TGGCGAGCAGGAGTATCAC-3’	RT-qPCR of CSF1
CSF1-R	5’-AGGTCTCCATCTGACTGTCAAT-3’	RT-qPCR of CSF1
CDIP1-F	5’-ATTGGCTTGATGAATTTCGTGC-3’	RT-qPCR of CDIP1
CDIP1-R	5’-GTGCGTCACATCCTTGAAGTC-3’	RT-qPCR of CDIP1
PEG10-F	5’-GAGCACCAGGGATTTCTCAGT-3’	RT-qPCR of PEG10
PEG10-R	5’-GGTAGTTGTGCATCAGGTAGTG-3’	RT-qPCR of PEG10
NGFR-F	5’-CCTACGGCTACTACCAGGATG-3’	RT-qPCR of NGF
NGFR-R	5’-CACACGGTGTTCTGCTTGT-3’	RT-qPCR of NGF
ICAM1-F	5’-ATGCCCAGACATCTGTGTCC-3’	RT-qPCR of ICAM1
ICAM1-R	5’-GGGGTCTCTATGCCCAACAA-3’	RT-qPCR of ICAM1
TRIL-F	5’-CCTCGGCGGCAACTTCATAA-3’	RT-qPCR of TRIL
TRIL-R	5’-AGAGCGGATCTGGTTGTACTG-3’	RT-qPCR of TRIL
PSG5-F	5’-CTGGAACCTGCCTATCACTGC-3’	RT-qPCR of PSG5
PSG5-R	5’-TGGTAGAGGTCCATCAGTTGTC-3	RT-qPCR of PSG5

ACTA2-F	5'-AAAAGACAGCTACGTGGGTGA-3'	RT-qPCR of ACTA
ACTA2-R	5'-GCCATGTTCTATCGGGTACTTC-3'	RT-qPCR of ACTA
SLC7A11-F	5'-TCTCCAAAGGAGGTTACCTGC-3'	RT-qPCR of SLC7A11
SLC7A11-R	5'-AGACTCCCCTCAGTAAAGTGAC-3'	RT-qPCR of SLC7A11
LIMK1-F	5'-CAAGGTGTACGGACAGGTTAGT-3'	RT-qPCR of LIMK1
LIMK1-R	5'-CCAATGCCAGGCTATCTCG-3'	RT-qPCR of LIMK1
HBEGF-F	5'-ATCGTGGGGCTTCTCATGTT-3'	RT-qPCR of HBEGF
HBEGF-R	5'-TTAGTCATGCCCAACTTCACTTT-3'	RT-qPCR of HBEGF
CALD1-F	5'-TGGAGGTGAATGCCCAGAAC-3'	RT-qPCR of CALD1
CALD1-R	5'-GAAGGCGTTTTTGGCGTCTTT-3'	RT-qPCR of CALD1
SORL1-F	5'-CAAGGTGTACGGACAGGTTAGT-3'	RT-qPCRof SORL1
SORL1-R	5'-CCAATGCCAGGCTATCTCG-3'	RT-qPCRof SORL1
GALNT7-F	5'-TGCTGGAGGAGATTCCCAGAA-3'	RT-qPCR of GALNT7
GALNT7-R	5'-GCACAGGATCATGGTAGGTGAA-3'	RT-qPCR of GALNT7
HIPK2-F	5'-AATAGAGCCGAGTTCCAACTGG-3'	RT-qPCR of HIPK2
HIPK2-R	5'-GTCTGCTCGTAAGGTAGGCTT-3'	RT-qPCR of HIPK2
SATB2--F	5'-GCAGTTGGACGGCTCTCTT-3'	RT-qPCR of SATB2
SATB2--R	5'-CACCTTCCCAGCTTGATTATTCC-3'	RT-qPCR of SATB2
KLHL29-F	5'-GCAGAGCGAAAGCGTTTACAG-3'	RT-qPCR of KLHL29
KLHL29-R	5'-CAGGTTCGACAGGACGAG-3'	RT-qPCR of KLHL29
EGFR-F	5'-AGGCACGAGTAACAAGCTCAC-3'	RT-qPCR of EGFR
EGFR-R	5'-ATGAGGACATAACCAGCCACC-3'	RT-qPCR of EGFR
Pri-miR-27a-F	5'-ATCACATTGCCAGGGATTTC-3'	RT-qPCR of mir-27a primary
Pri-miR-27a-R	5'-GGCGGAACTTAGCCACTGT-3'	RT-qPCR of mir-27a primary
Pre-miR-27a-F	5'-GCAGGGCTTAGCTGCTTG-3'	RT-qPCR of mir-27a precursor
Pre-miR-27a-R	5'-GGCGGAACTTAGCCACTGT-3'	RT-qPCR of mir-27a precursor

miR-27a-sense	5’-AGGGCTTAGCTGCTTGTGAGCA-3’	RT-qPCR of mir-27a mature
Universal primer	5’-GCGAGCACAGAATTAATACGAC-3’	RT-qPCR of mir-27a mature
U6-F	5’-CTCGCTTCGGCAGCACA-3’	Internal reference for small RNA
U6-R	5’-AACGCTTCACGAATTTGCGT-3’	Internal reference for small RNA
miR-27a -mimics	5’-UUCACAGUGGCUAAGUUCCGCGGAACUU AGCACUGUGAAUU-3’	transfection
mimics NC	5’ UUGUACUACACAAAAGUACUG-3’	transfection
miR-27a-inhibitor	5’-GCGGAACUUAGCCACUGUGAA-3’	transfection
Inhibitor NC	5’-CAGUACUUUUGUGUAGUACAA-3’	transfection
sgRNA30-F	5’-caccgGGAGGTGAGGGCCTGGGGGG-3’	CRISPR-Cas9 KO construction for miR-27a
sgRNA30-R	5’-aaacCCCCCCAGGCCCTCACCTCC-3’	
sgRNA31-F	5’-caccgGCAGCTAAGCCCTGCTCCTC-3’	CRISPR-Cas9 KO construction for miR-27a
sgRNA31-R	5’-aaacGAGGAGCAGGGCTTAGCTGCC-3’	
sgRNA32-F	5’-caccgACACCAAGTCGTGTTCACAG-3’	CRISPR-Cas9 KO construction for miR-27a
sgRNA32-R	5’-aaacCTGTGAACACGACTTGGTGT-3’	
