# Supplementary material for: A functional SNP rs895819 on pre-miR-27a is associated with bipolar disorder by targeting NCAM1
Source: Commun Biol. 2022 Apr 4;5:309. doi: 10.1038/s42003-022-03263-6 (PMC8980034; doi:10.1038/s42003-022-03263-6)
Supplement: Supplementary file 3 — Description of Additional Supplementary Files [file 42003_2022_3263_MOESM3_ESM.pdf]

Description of Additional Supplementary Files

File name: Supplementary Data 1
Description: Differentially expressed genes in U251 WT and KO

File name: Supplementary Data 2
Description: RT-qPCR validation of up-regulated genes in miR-27a knockout

File name: Supplementary Data 3
Description: Expression of WT and mutant miR-27a in NPCs

File name: Supplementary Data 4
Description: The qpcr data of miR-27a in NPC and U251

File name: Supplementary Data 5
Description: The data of luciferase reporter assay

File name: Supplementary Data 6
Description: The migrating cell number of control and mutant allele
