# Supplementary material for: A functional SNP rs895819 on pre-miR-27a is associated with bipolar disorder by targeting NCAM1
Source: Commun Biol. 2022 Apr 4;5:309. doi: 10.1038/s42003-022-03263-6 (PMC8980034; doi:10.1038/s42003-022-03263-6)
Supplement: Supplementary file 10 — Reporting Summary [file 42003_2022_3263_MOESM10_ESM.pdf]

nature portfolio

Corresponding author(s): Yifeng Yang

Last updated by author(s): 2022-2-12

Reporting Summary

Nature Portfolio wishes to improve the reproducibility of the work that we publish. This form provides structure for consistency and transparency in reporting. For further information on Nature Portfolio policies, see our Editorial Policies and the Editorial Policy Checklist.

Statistics

For all statistical analyses, confirm that the following items are present in the figure legend, table legend, main text, or Methods section.

n/a	Confirmed	
☐	☒	The exact sample size (n) for each experimental group/condition, given as a discrete number and unit of measurement
☐	☒	A statement on whether measurements were taken from distinct samples or whether the same sample was measured repeatedly
☐	☒	The statistical test(s) used AND whether they are one- or two-sided<br>*Only common tests should be described solely by name; describe more complex techniques in the Methods section.*
☐	☒	A description of all covariates tested
☐	☒	A description of any assumptions or corrections, such as tests of normality and adjustment for multiple comparisons
☐	☒	A full description of the statistical parameters including central tendency (e.g. means) or other basic estimates (e.g. regression coefficient) AND variation (e.g. standard deviation) or associated estimates of uncertainty (e.g. confidence intervals)
☐	☒	For null hypothesis testing, the test statistic (e.g. F, t, r) with confidence intervals, effect sizes, degrees of freedom and P value noted<br>*Give P values as exact values whenever suitable.*
☐	☒	For Bayesian analysis, information on the choice of priors and Markov chain Monte Carlo settings
☐	☒	For hierarchical and complex designs, identification of the appropriate level for tests and full reporting of outcomes
☐	☒	Estimates of effect sizes (e.g. Cohen's d, Pearson's r), indicating how they were calculated

Our web collection on statistics for biologists contains articles on many of the points above.

Software and code

Policy information about availability of computer code

Data collection	SNPedia miRNA project; Hapmap Phase III ; TargetScan Human 7.1; miRDB
Data analysis	Shesis; G power 3.0; Graphpad Prism 9.0

For manuscripts utilizing custom algorithms or software that are central to the research but not yet described in published literature, software must be made available to editors and reviewers. We strongly encourage code deposition in a community repository (e.g. GitHub). See the Nature Portfolio guidelines for submitting code & software for further information.

Data

Policy information about availability of data

All manuscripts must include a data availability statement. This statement should provide the following information, where applicable:

- Accession codes, unique identifiers, or web links for publicly available datasets
- A description of any restrictions on data availability
- For clinical datasets or third party data, please ensure that the statement adheres to our policy

Data is contained within the article and supplementary materials. The NGS gene analysis data were deposited in the NCBI GEO database (https://www.ncbi.nlm.nih.gov/geo), under the accession code SRP146080.

Field-specific reporting

Please select the one below that is the best fit for your research. If you are not sure, read the appropriate sections before making your selection.

☒ Life sciences ☐ Behavioural & social sciences ☐ Ecological, evolutionary & environmental sciences

For a reference copy of the document with all sections, see nature.com/documents/nr-reporting-summary-flat.pdf

Life sciences study design

All studies must disclose on these points even when the disclosure is negative.

Sample size	in a Chinese sample of 528 schizophrenia subjects, 528 bipolar disorder subjects and 528 control subjects.
Data exclusions	10 SNPs located in psychiatry susceptible loci, whose minor allele frequency was larger than 0.05 in Chinese Han population.
Replication	Replicate using three cell lines (U251MG, SH-SY5Y, Neural progenitor cell),<br>using three more different methods(RNAseq, QPCR, Western blotting, Luciferase Reporter Assay)
Randomization	Schizophrenia and bipolar disorder patients were diagnosed on the basis of DSM-III-R criteria . Each patient was assessed by at least two psychiatrists independently according to the case records and interviews.
Blinding	blinding test designed in RNAseq, WB, QPCR

Reporting for specific materials, systems and methods

We require information from authors about some types of materials, experimental systems and methods used in many studies. Here, indicate whether each material, system or method listed is relevant to your study. If you are not sure if a list item applies to your research, read the appropriate section before selecting a response.

Materials & experimental systems

n/a	Involved in the study	
☐	☒	Antibodies
☐	☒	Eukaryotic cell lines
☒	☐	Palaeontology and archaeology
☒	☐	Animals and other organisms
☐	☒	Human research participants
☒	☐	Clinical data
☒	☐	Dual use research of concern

Methods

n/a	Involved in the study	
☒	☐	ChIP-seq
☒	☐	Flow cytometry
☒	☐	MRI-based neuroimaging

Antibodies

Antibodies used	ICAM1 antibody （ab53013, abcam), NCAM1 antibody (14255-1-AP, ProteinTech) ; β-actin (#4970, CST);α-tubulin (#9099,CST)<br>Nestin,(#4760 , CST); Sox, SC-365823 ,santa cruz; or Tuj1, GTX631836 from GeneTex and MAP2,17490-1-AP from Proteintech
Validation	ICAM1 Rabbit monoclonal [EP1442Y] to ICAM1,Suitable for: WB, IHC; Knockout validated ;Reacts with: Human<br>NCAM1 antibody, 14255-1-AP, Proteintech, Rabbit polyclonal, suitable for :FC, IHC, WB, ELISA; KO,kD validated; React with H,M,R .<br>β-actin antibody, #4970,CST,Rabbit mAb,suitable for WB,IHC,IF,F. React with H M R Mk B Pg.<br>α-tubulin antibody,#9099,CST, Rabbit mAb,suitable for WB,React with H M R Mk Dm Z B Pg.<br>Nestin antibody，#4760,CST,Mouse mAb,suitable for IHC,IF, React with H R<br>Tuj1 antibody,GTX631836,GeneTex, Mouse mAb,suitable for WB, ICC/IF, IHC-P, IHC-Fr, IP ,React with Human, Mouse, Rat, Fish.<br>Sox2 antibody,SC-365823,Santa Cruz, Mouse mAb,suitable for WB, ICC/IF,IP,IF,React with mouse, rat ,human.<br>MAP2 antibody,17490-1-AP,Proteintech，polyclonal antibody，suitable for FC, IF, IHC, IP, WB,ELISA.React with H M R.

Eukaryotic cell lines

Policy information about cell lines

Cell line source(s)	ATCC

Authentication	U251-MG,SH-SY5Y, NPC were authenticated at VivaCell Shanghai using short tandem repeat analysis.
Mycoplasma contamination	no mycoplasma contamination
Commonly misidentified lines (See ICLAC register)	no

Human research participants

Policy information about studies involving human research participants

Population characteristics	1 Study Population<br>We recruited 1,584 patients from southern China, including 528 schizophrenia patients with a mean age of onset at 47.3 ± 13.4 (52.3% male), 528 bipolar disorder patients with a mean age of onset at 41.1 ± 12.8 (55.3% male) and 528 unrelated healthy individuals with a mean age of 42.72 ± 13.1 (43.6% male) as the control group.
Recruitment	Schizophrenia and bipolar disorder patients were diagnosed on the basis of DSM-III-R criteria . Each patient was assessed by at least two psychiatrists independently according to the case records and interviews.
Ethics oversight	A standard informed consent was signed by each participant and reviewed and approved by the Shanghai Ethical Committee of Human Genetic Resources.

Note that full information on the approval of the study protocol must also be provided in the manuscript.
